# Identification of potential light deficiency response regulators in endangered species *Magnolia sinostellata*

**DOI:** 10.1038/s41598-022-25393-x

**Published:** 2022-12-29

**Authors:** Danying Lu, Bin Xu, Qin Yu, Zhigao Liu, Mingjie Ren, Yaling Wang, Shouzhou Zhang, Chao Wu, Yamei Shen

**Affiliations:** 1 College of Landscape and Architecture, Zhejiang Agriculture and Forestry University, Hangzhou, 311300 Zhejiang China; 2 Xi’an Botanical Garden of Shanxi Academy of Science, Xi’an , 710061 Shanxi China; 3 Fairy Lake Botanical Garden, Shenzhen, 518004 Guangdong China

**Keywords:** Ecology, Genetics, Molecular biology, Plant sciences

## Abstract

*Magnolia sinostellata* is one of the endangered species in China and largely suffers light deficiency stress in the understory of forest. However, the weak light response molecular mechanism remains unclear. More importantly, hub genes in the molecular network have not been pinpointed. To explore potential regulators in the mechanism, weighted gene co-expression network analysis (WGCNA) was performed to analysis the trancriptome data of *M. sinostellata* leaves subjected to weak light with different time points. Gene co-expression analysis illustrated that module 1, 2 and 3 were closely associated with light deficiency treatment, which. Gene ontology and KEGG analyses showed that genes in module 1 mainly participated in amino and nucleotide metabolism, module 2 mostly involved in carbon fixation and module 3 mostly regulated photosynthesis related pathways, among which 6, 7 and 8 hub genes were identified, respectively. Hub genes isoform_107196 in module 1 and isoform_55976 in module 2 were unique to *M. sinostellata*. This study found that light deficiency inhibited photosynthesis and stress tolerance, while improved carbon metabolism and flowering related pathways in *M. sinostellata*, which can impact its accumulation reserves of growth and reproduction in the next season. In addition, key shade response regulators identified in this study have laid a firm foundation for further investigation of shade response molecular mechanism and protection of other shade sensitive plants.

## Introduction

Records on Magnoliaceae plants can be traced back to the Mesozoic era. Until now, many Magnoliaceae plants have been favored by people for their ornamental characteristics. However, with the changes of the climate, forest community composition, and the succession of forests, deciduous Magnoliaceae species are facing endangerment in the wild^1^. Nowadays, the growth and distribution of endangered species of Magnoliaceae are mostly confined to narrow areas. In natural condition, Magnoliaceae plants was mainly adapted to coniferous and broad-leaved mixed communities, while declined to evergreen broad-leaved communities^2–4^. *Magnolia sinostellata* is an endangered species belonging to family Magnoliaceae. It is a deciduous shrub, listed in *the Red List of Magnoliaceae* since 2016, which can grow to 3 m in height and blossom in early spring (February and March) in subtropical regions^5^. *M. sinostellata* is endemic to a narrow area of China (mainly distributed in Jingning, Wenzhou county, Zhejiang Province), with extremely small population. *M. sinostellata* is a sun-loving plant, and it largely grows under canopy shade or nearby the brook of the north slope in its natural habitats as its seedling growth requires mild degree of shading^5^. Moreover, pre-investigations indicated that *M. sinostellata* mainly distributed in coniferous forests, sparsely distributed in broad-leaved forests and mixed forests^4^. As the dominant tree species in the upper layer of different forests are different, the light condition in the under story of different forest type can be varying. For instance, the light intensity of coniferous communities is higher than that of evergreen broad-leaved communities^6^. Previous research has simulated light intensities under different forest types using black shade net, finding that heavy shading (equivalent to the light intensity under a closed broad-leaved forest) suppressed *M. sinostellata* seedlings by impacting their chlorophyll metabolic pathway, photosynthesis and antioxidant systems^7^. These findings suggest that alternation in light environment caused by upper canopy shade might be the key factor that limiting the distribution and population renewal of *M. sinostellata*. Canopy shading of evergreen trees in the upper layer changes the light intensity as well as the light quality (ratio of red/far-red light, R/FR), which will affect the growth of deciduous plants in the understory^6,8–12^. As light intensity and light quality that reaches chloroplasts is essential for activation of photosynthesis, the primary effect of shading on plants is to affect their photosynthesis^13^. The R/FR ratio of natural light in undergrowth declined as photosynthetic pigments of vegetative canopy absorb red light^14^. Low R/FR ratio changes chlorophyll content and directly affect plant photosynthetic capacity^15^. Leaf occlusion of upper layer can also reduce light intensity in the undergrowth, which directly affects photosynthesis via inhibiting the expression of photosynthetic proteins and altering chloroplast ultrastructure^16,17^. Under weak light conditions, light captured by light harvesting complex (LHC) is limited^18^. In *Zea may*, photochemical quenching coefficient (qp) and effective quantum yield of PSII photochemistry (ΦPSII) were significantly reduced under low light^19^. Due to the defect in PSII, Electron transport rate through PS I (ETR I) is significantly blocked in Rice^20^. Thus, nicotinamide adenine dinucleotide phosphate (NADPH) generated for further CO_2_ assimilation is insufficient. Furthermore, the activity of ribulose 1,5-bisphosphate carboxylase/oxygenase (Rubisco), key enzyme that transforms CO_2_ into carbohydrates^21^, is repressed and its content is decreased under shade^22^, indicating that carbon assimilation was suppressed under shading in shade sensitive plants such as rice^23^, *Cucumis sativus*^24^ and maize^25^. While moderate shading increased both photosynthesis Rubisco activity in *C. sativus*^24^ and *Sargassum henslowianum*^26^, thereby enhancing carbon assimilation and stress tolerance. Shade tolerant plants *Arachis hypogaea*^27^ and *Quercus robur*^28^ can maintain rubisco activity and photosynthesis efficiency under shading environment.

In plant, the rate of photosynthetic CO_2_ fixation determines the rate of starch and sucrose synthesis and other carbon metabolism^29^. Sucrose generated via the Calvin cycle, which further secreted into reservoir organs to participate starch synthesis^30^. The limitation of activity of sucrose phosphate synthase and sucrose synthase in response to shading indicates that its starch and sucrose metabolism was also inhibited^31^. For shade sensitive plants, with the increase of shading degree, the carbon assimilation and metabolism capacities of seedlings decreases^32^, water usage and transpiration are also affected^33^, which affect plant growth and community regeneration. In contrast, the expression of genes regulating photosynthesis and carbon metabolism in shade tolerant plant *Solidago canadensis* increased to maintain carbon fixation under weak light condition^34^.

To compete for sunlight, under vegetative shading, elongation response was triggered and flowering process was dramatically accelerated in shade-avoiding Arabidopsis^35^, cucumber^36^ and tomato^37^, etc. The change in light quality under canopy shade (low R/FR ratio) could be sensed by phytochrome photoreceptors^14^, which activate classical shade avoidance response subsequently. The low light intensity can also be detected in plants and cause elongation phenotype in shade sensitive soybean^38^ and *Brassica*^39^. Whereas, these responses can be detrimental, especially for agricultural crops and ornamental plants, because the reallocation of resources into vegetative growth reduced reproductive growth and crop yield^40^. In ornamental plants, shading inevitably altered their flower period, flower quality and numbers, which eventually destroyed their ornamental and economic values. For instance, weak light dramatically reduced flower quality, alkaloid yield and seed number of *Papaver somniferum*^41^. Although shading accelerated flowering in some plants, while in *Paeonia lactiflora*, shading led to delayed flowering date, as well as reduced flower fresh weight and faded flower color^11^. These studies showing that, even in shade sensitive species, the shade response can be species–dependent. In our previous study, we found that shading caused light deficiency boosted chlorophyll degradation, leaf chlorosis and senescence in *M. sinostellata*^42^, which also found in rice^43^ and *Camellia sinensis*^44^. While accelerated leaf abscission under weak light was only found in *M. sinostellata* and severely impaired its growth, which has not been reported in other species^42^. However, as the impact of light deficiency on reproductive growth of *M. sinostellata* and other vital metabolic mechanism still unknown, these findings were not able to explain how low light affects the population renewal of *M. sinostellata*, which eventually limiting its distribution in the wild. Furthermore, despite the light environment under canopy shade containing reduced light intensity and inconsistent light quality, in order to reduce the confounding factors, many studies mainly focused on impact of weak light on plant growth^45–47^. Accordingly, in this study, we continued investigating the effect of light deficiency on *M. sinostellata*.

Although numerous low-light responsive pathways, transcription factors, and R-genes has been identified in *M. sinostellata*, the hub genes in its light deficiency remains unknown and the weak light response mechanism still obscure. In this study, co-expressed modules and hub genes in transcriptomes of light deficiency treated and untreated *M. sinostellata* leaves were pinpointed via weighted gene co-expression network analysis (WGCNA). The functions of these genes were analyzed via KEGG, Gene Ontology (GO) and National Center for Biotechnology Information (NCBI), which shed more light on the mystery of light deficiency response mechanism of *M. sinostellata*. This study forms a theoretical basis for protection and conservation of *M. sinostellata* and other shade sensitive woody plants.

## Results

### Identification of the light deficiency-responsive core DEGs in *M. sinostellata*

Significant morphological and physiological changes were observed in *M. sinostellta*, relative to control group (CK), under light deficiency condition. The seedlings in CK group grew well and leave were fully expanded during the experiment. Five days after being subjected to light deficiency condition, seedlings began to wilt. By 15 d of low light treatment, the wilting of the seedling aggravated and severe leaf abscission was observed (Fig. S1). Moreover, the net photosynthetic rate (*Pn*) and photochemical efficiency (*Fv/Fm*) declined obviously during the treatment, indicating that photosynthesis ability of *M. sinostellata* impaired^42^.

In order to gain more insight into the weak light response mechanism, transcriptome sequencing was performed. A total of 181, 902 genes were detected via transcriptome sequencing, and the average comparison rate with the compared gene set was 80.49%. 246,481 non-redundant transcript sequences were obtained. The total length of these sequences is 270,112,156, the longest sequence is 20445 bp, the shortest sequence is 199 bp, and the average sequence length is 3420.95 bp. In this study, we analyzed the global gene expression profiles of *M. sinostellata* for light deficiency response using five datasets, including M-D0, CK-D5, LT-D5, CK-D15 and LT-D15, the details of which are shown in Table S1. Each dataset contains 3 replicates. Correlation analysis was performed among these 15 samples, which showed good reproductivity in the same group and a significance difference between control groups and shade treated groups (Fig. S2). This analysis suggests that these samples can be used for subsequent screening and analysis.

**Figure 1:**
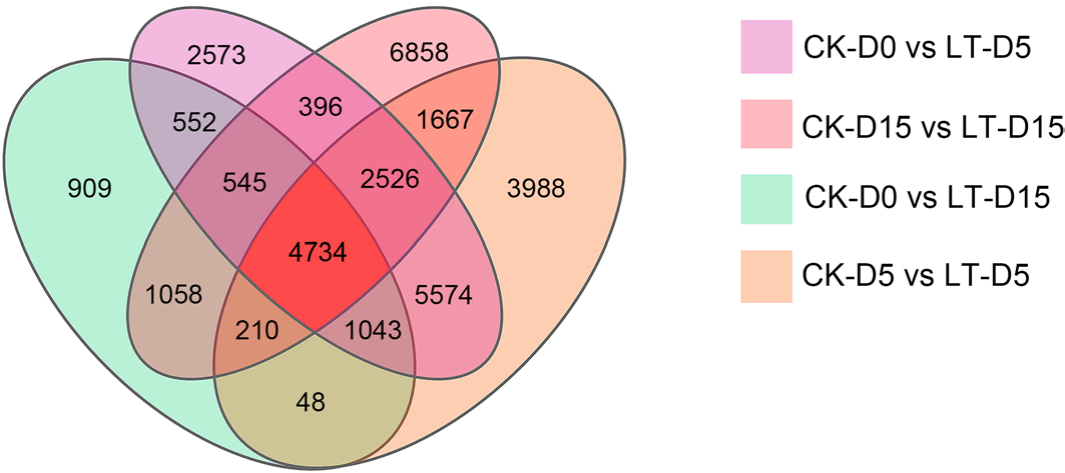
Venn diagram of differential expression genes in treated and control groups in *M. sinostellata* (R software, version 4.0.3, www.gnu.org/software/r/). Venn diagram showing 4734 core low light responsive DEGs among treated and control groups used in this study.

**Figure 2:**
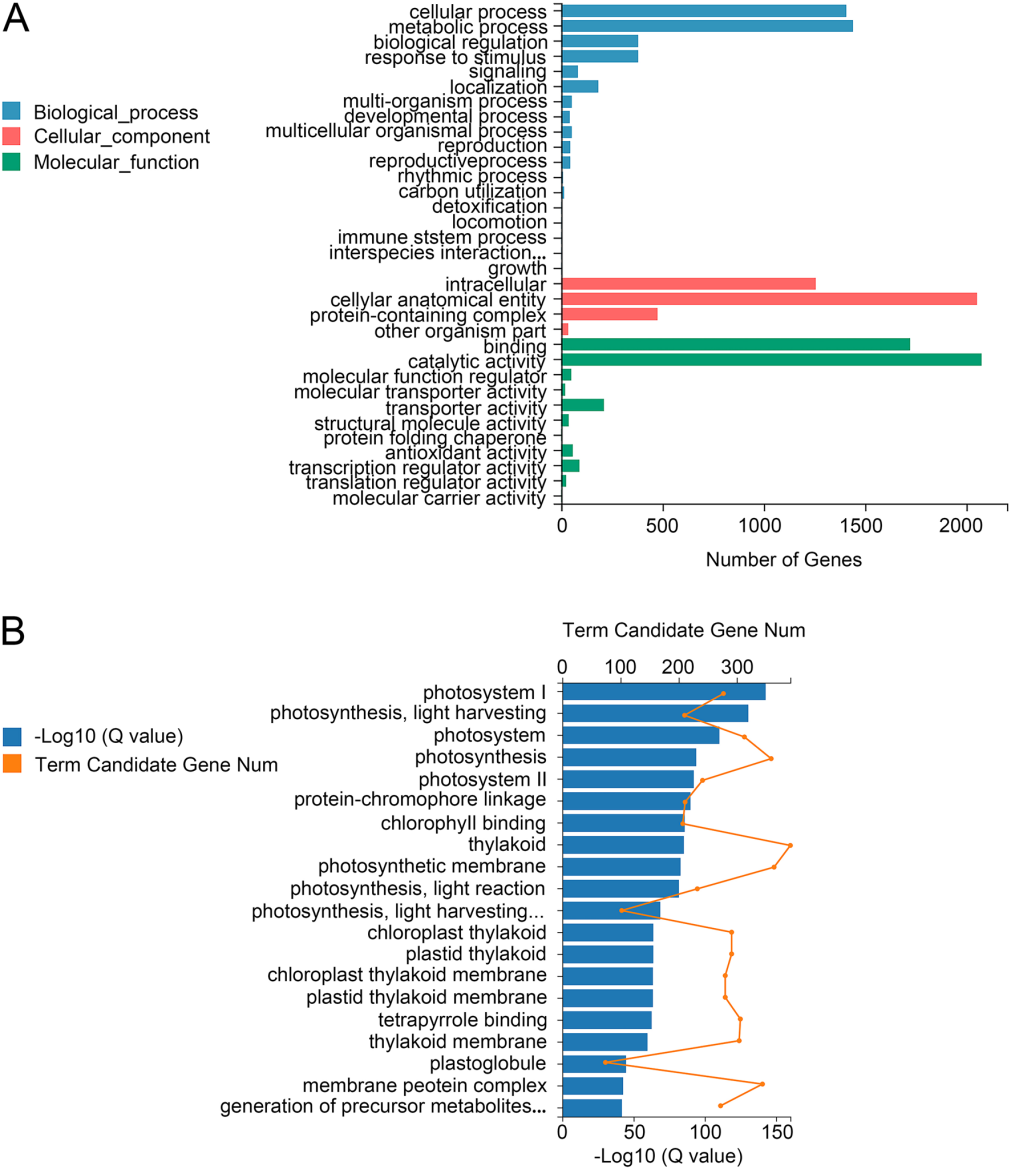
Gene ontology (GO) analysis of 4734 core DEGs (R software, version 4.0.3, www.gnu.org/software/r/). (**A**) GO classification of 4734 core DEGs. 4734 core DEGs were divided into three main categories and 36 subgroups. (**B**) GO enrichment of 4734 core DEGs. The top 20 GO Terms with the smallest Qvalue were selected to plot the chart.

**Figure 3:**
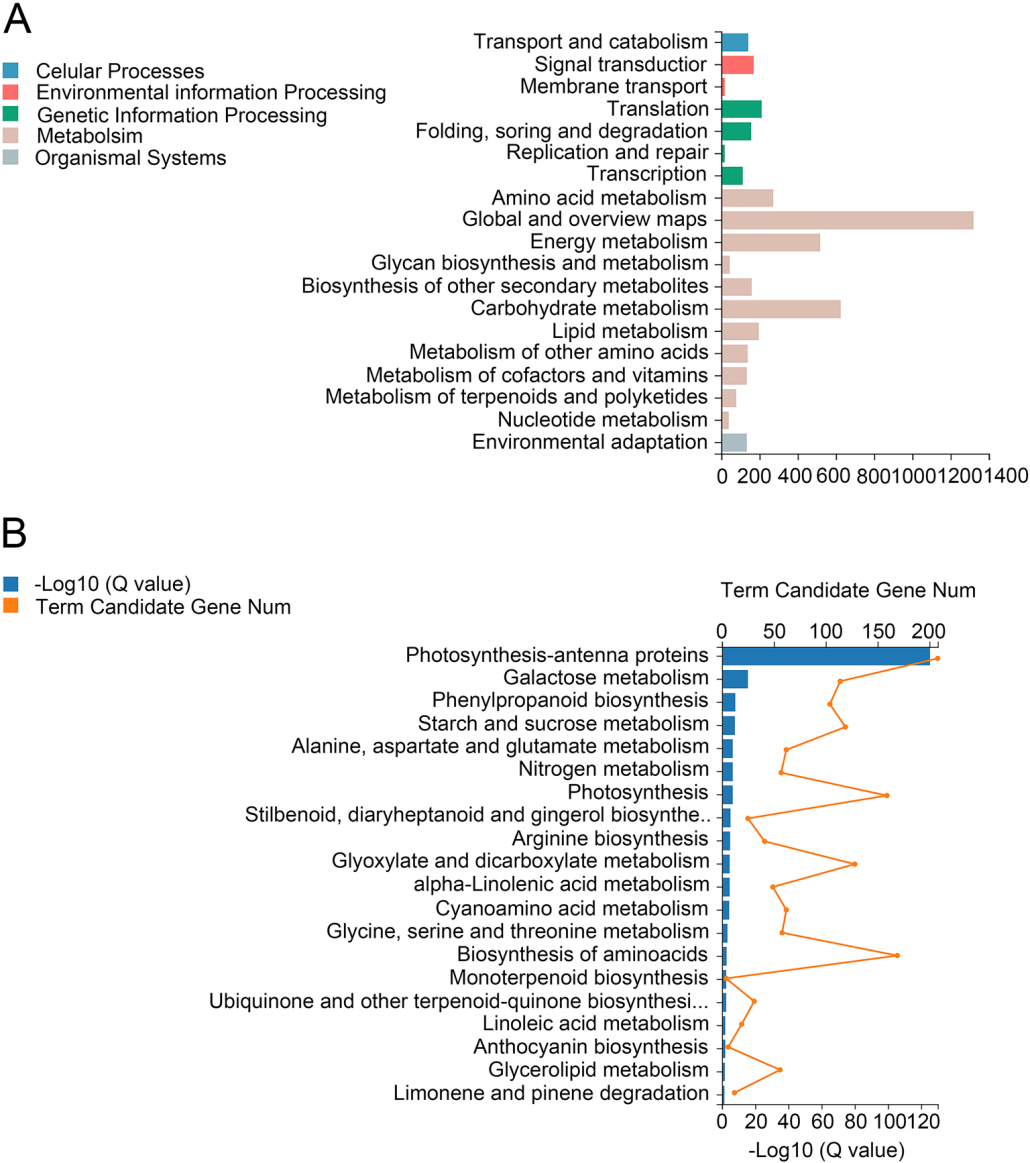
KEGG analysis of core DEGs in *M. sinostellata* (R software, version 4.0.3, www.gnu.org/software/r/). (**A**) KEGG classification of core DEGs. The metabolism pathways contributed by core DEGs were divided into five groups and 19 subgroups. (**B**) KEGG enrichment of 4734 core DEGs. The top 20 KEGG pathways with the smallest Qvalue were selected to plot the chart, among which 'Photosynthesis-antenna proteins' was the most enriched pathway.

Pairwise comparison identified 17,943, 9102, 19,790 and 17,994 in M-D0 vs LT-D5, M-D0 vs LT-D15, CK-D5 vs LT-D5 and CK-D15 vs LT-D15 comparison groups, respectively (Log2FC > 1, Qvalue < 0.05). 4734 core DEGs were found in their cross-compared Venn diagrams, which were significantly induced or suppressed during the experiment (Fig. 1, Table S2). Then, we characterized these 4734 core DEGs to get more insight into their contributing molecular pathways. First, 4734 DEGs were subjected to GO analysis, which were classified into three groups and 33 subgroups (Fig. 2A). The biological process group can be divided into 18 subgroups, among which cellular process and metabolic process were the top two sub-groups involved the most genes. Four subgroups were related to cellular component, among which cellular anatomical entity and intracellular were the main subgroups involved most genes. Eleven subgroups constitute the molecular function group, and the catalytic activity and binding involved the most genes. GO enrichment analysis showed that most significant GO terms are related to photosynthesis. The top five enriched GO terms were (GO:0009522); photosynthesis, light harvesting (GO:0009765); photosystem (GO:0009521); photosynthesis (GO:0015979) and photosystem II (GO:0009523) (Fig. 2B). Then, we performed KEGG analysis to explore contributing pathways of 4734 DEGs (Fig. 3A). For KEGG classification, genes were annotated into five groups and 19 subgroups. Among metabolism group, global and overview maps, carbohydrate metabolism and energy metabolism subgroups were significantly enriched. Photosynthesis-antenna proteins was the most significantly enriched KEGG pathway. The top five frequently enriched pathways were as follows: photosynthesis-antenna proteins, galactose metabolism, phenylpropanoid biosynthesis, starch and sucrose metabolism and alanine, aspartate and glutamate metabolism (Fig. 3B).

### WGCNA of core light deficiency responsive DEGs in *M. sinostellata*

**Figure 4:**
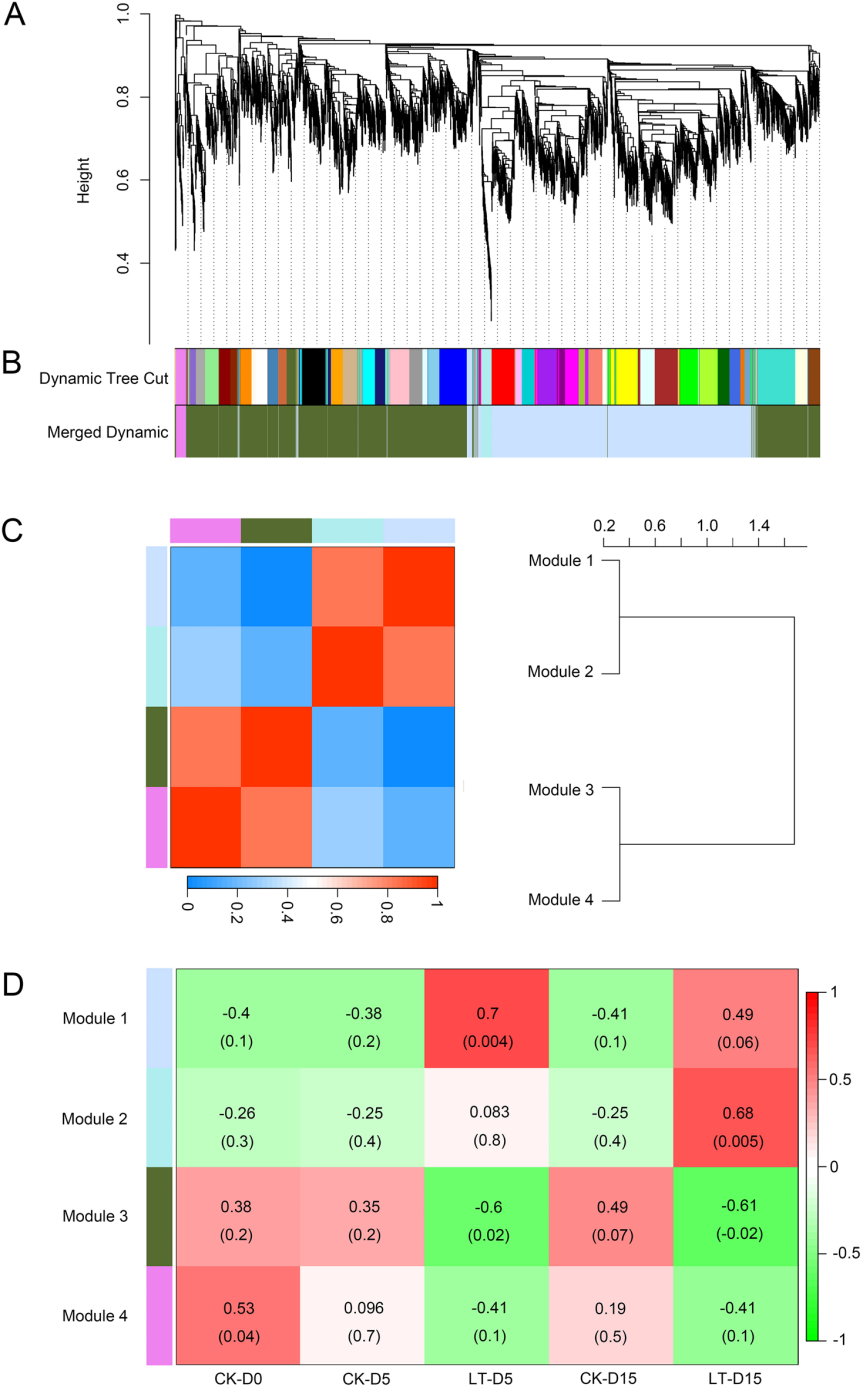
WGCNA module identification of 4734 core DEGs in *M. sinostellata* (R software, version 4.0.3, www.gnu.org/software/r/). (**A**) hierarchical clustering tree of 4734 core DEGs in *M. sinostellata*. (**B**) Dynamic tree cut and merged modules. (**C**) Dendrogram heatmap of four merged modules. (**D**) Module-trait relationship heat map. the correlation of the identified modules in control and shade treated groups. Red and green color illustrated positive and negative correlation with gene expression, respectively.

**Figure 5:**
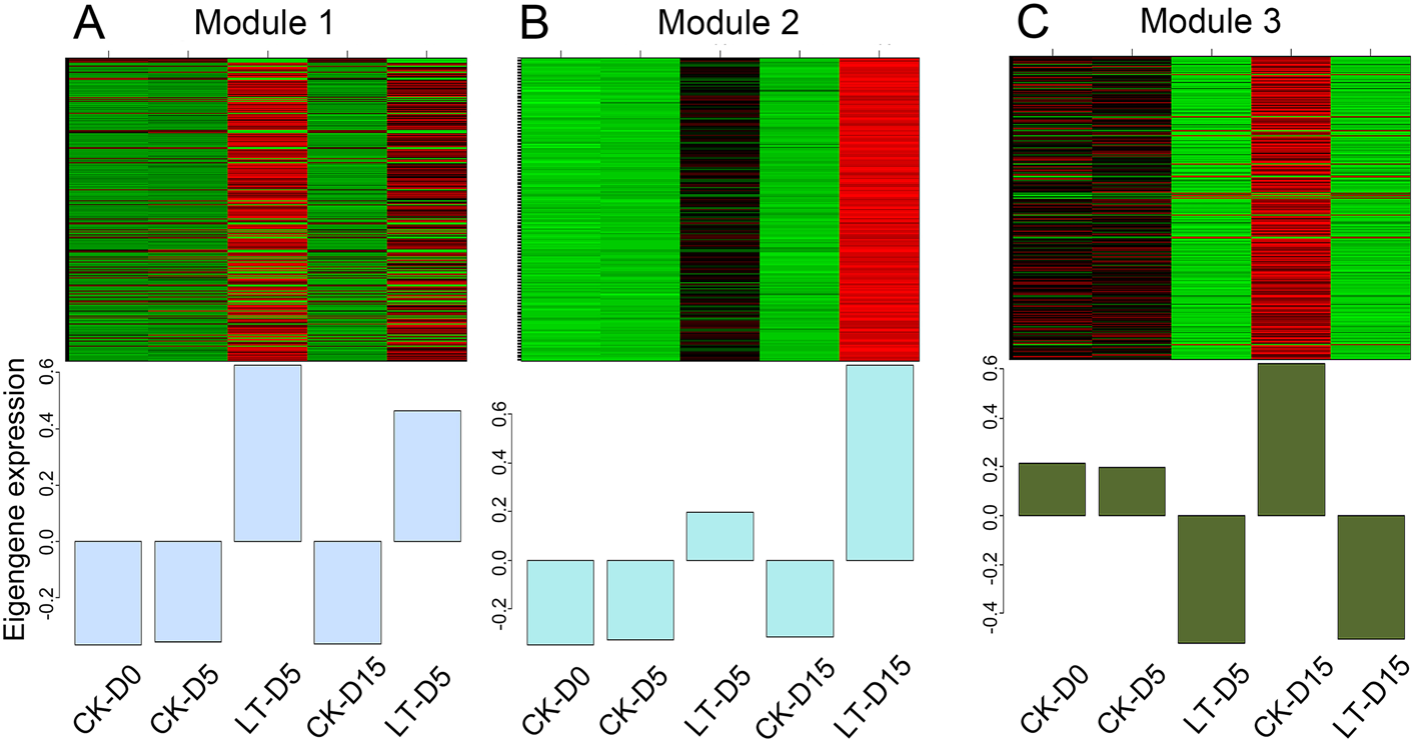
Eigengene expression pattern of key light deficiency associated modules (R software, version 4.0.3, www.gnu.org/software/r/). (**A**) Eigengene expression pattern of Module 1. (**B**) Eigengene expression pattern of Module 2 module. (**C**) Eigengene expression pattern of Module 3. Eigengene expression pattern is the optimal tool to summarize expression pattern of specific module.

To identify key shade responsive genes in *M. sinostellata*, WGCNA was performed to analysis 4734 core DEGs, which can identify modules of highly related genes. The selection of an optimal soft threshold power is an essential step to construct WGCNA. A network topology research of 1–20 was executed, and the scale independence and the average connectivity of the WGCNA relative equilibrium were determined. Threshold 18 was selected to construct a hierarchical clustering tree of DEGs (Fig. 4A). MEDiss Thres was set to 0.22 to merge similar modules and 4 modules was generated (Fig. 4B). Genes in grey module that cannot be assigned to any modules were not analyzed in the further study. Four different modules were generated by WGCNA including module 1, module 2, module 3 and module 4, which including 2008, 75, 2481, and 71 DEGs, respectively (Table 1). To identify co-expression similarity of modules, characteristic genes were calculated and clustered according to their correlation (Fig. 4C). We found that these 4 modules are divided into two categories: the first included module 1 and module 2 modules; the second included module 3 and module 4. Gene modules of the same category may have similar functions or contributing to the same regulatory mechanism. To investigate modules associated with light deficiency treatment, we plotted module-trait relationship heat map (Fig. 4D). This result shows that module 1 positively correlated with two weak light-treated groups (LT-D5, r = 0.7; LT-D15, r = 0.49) but negatively correlated with control groups (M-D0, r = − 0.40; CK-D5, r = − 0.38; CK-D15, r = − 0.41). Similarly, module 2 had a strong correlation with LT-D15 (r = 0.68), while had a negative correlation with M-D0 (r = − 0.26), CK-D5(r = − 0.25) and CK-D15 (r = − 0.25). In contrast, module 3 showed a positive correlation with control groups (M-D0, r = 0.38; CK-D5, r = 0.35; CK-D15, r = 0.49) but illustrated a significant negative correlation with treated groups (cor < − 0.5, p < 0.05). However, genes in module 4 showed no significant correlation with treated groups or control groups. These results suggest that DEGs in module 1 and module 2 mainly up-regulated and DEGs in module 3 down-regulated under light deficiency treatment. These results showed that module 1, module 2 and module 3 were significantly correlated with light deficiency treatment. To obtain further understand of the expression pattern of these three modules, heat maps of gene expression for these modules were generated along with eigengene expression values (Fig. 5). We observed that genes inmodule 1 were significantly responsive to light deficiency and mainly up regulated. In module 2, genes were slightly induced in LT-D5 while markedly up-regulated in LT-D15. In contrast, genes in module 3 mainly downregulated during the experiment.

**Table 1:** Details of four detected modules.

Module	Gene number
Module 1	2008
Module 2	75
Module 3	2481
Module 4	71

### GO and KEGG pathways analysis of DEGs in key modules in *M. sinostellata* associated with weak light

**Figure 6:**
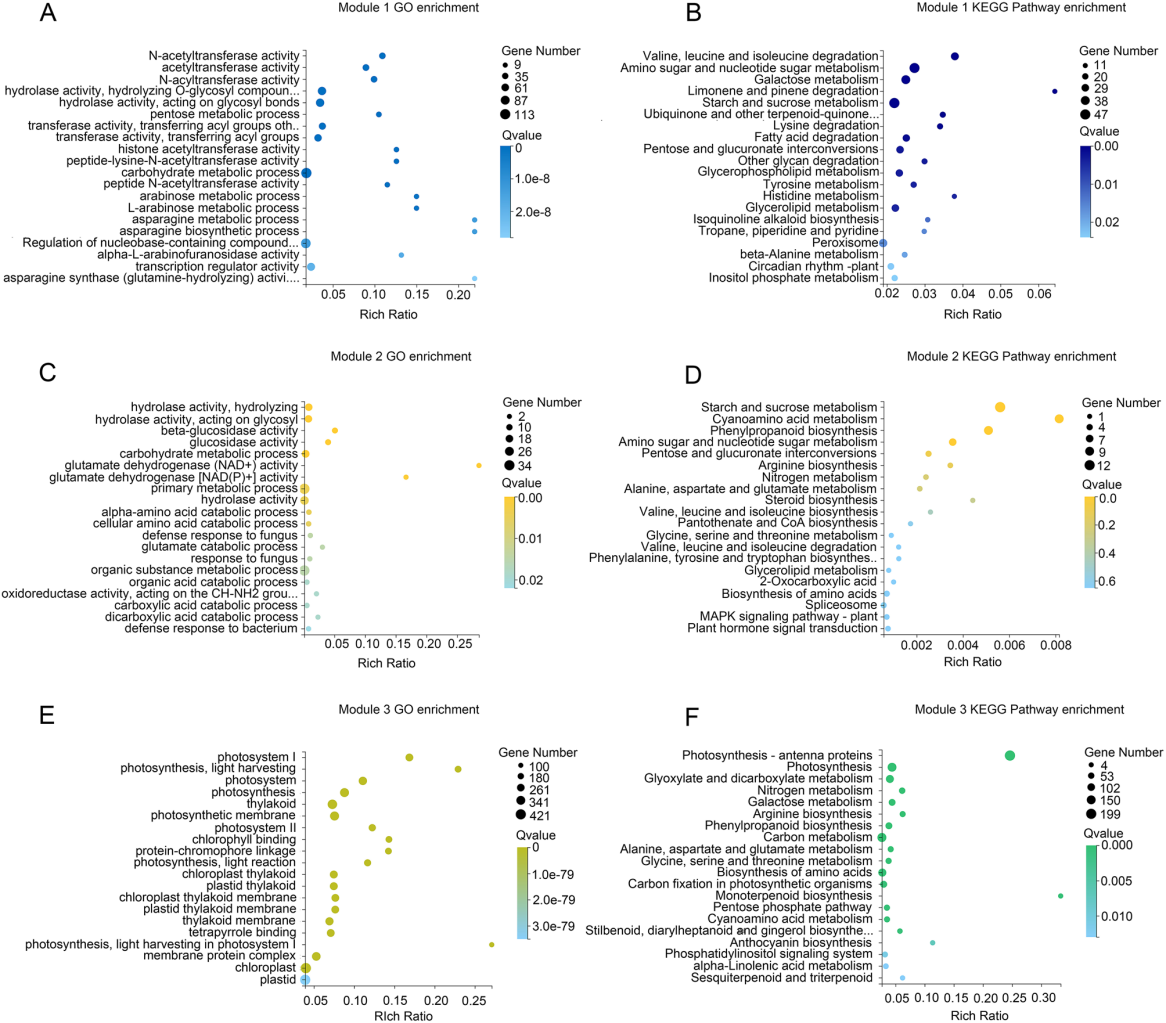
GO and KEGG enrichment analysis of Module 1, Module 2 and Module 3 (R software, version 4.0.3, www.gnu.org/software/r/). (**A**) GO enrichment analysis of Module 1. (**B**) KEGG pathway enrichment analysis of Module 1. (**C**) GO enrichment analysis of Module 2. (**D**) KEGG pathway enrichment analysis of Module 2. (**E**) GO enrichment analysis of Module 3. (**F**) KEGG pathway enrichment analysis of Module 3. The top 20 GO terms or KEGG pathways with the smallest Qvalue were selected to plot these charts.

The functions of genes under light deficiency treatment in specific modules and its contributing regulatory pathways were revealed by GO and KEGG analysis. GO analysis suggested that ‘N-acetyltransferase activity’ and ‘acetyltransferase activity’ as the most significantly enriched GO terms in module 1, which means genes in this module mainly response to weak light by regulating acetyltransferase activity (Fig. 6A). KEGG analysis in module 1 identified ‘Valine, leucine and isoleucine degradation’ and ‘Amino sugar and nucleotide sugar metabolism’ as the most enriched regulatory pathways (Fig. 6B). Concerning module 2, GO analysis displayed that ‘hydrolase activity’ and ‘hydrolyzing O-glycosyl compound’ were the top two enriched GO terms indicating that these genes regulating hydrolase-related metabolism (Fig. 6C). KEGG analysis demonstrated that ‘Starch and sucrose metabolism’ pathway was appreciably influenced by low light (Fig. 6D). These results matched the results of GO and KEGG analysis of 4734 core DEGs, indicating that these results are reliable. In module 3, GO analysis indicated that enriched GO terms of cellular component, biological process and molecular function were mainly related to photosynthesis, among which the top five GO terms were ‘photosystem I’, ‘photosynthesis, light harvesting’, ‘photosystem’, ‘photosynthesis’ and ‘thylakoid’ (Fig. 6E). KEGG analysis showed that ‘Photosynthesis-antenna proteins’ and ‘Photosynthesis’ were the most enriched pathways (Fig. 6F). These indicate that genes in module 3 mainly involved in photosynthesis.

### Hub genes identification in key Co-Expressed modules in *M. sinostellata* under light deficiency

**Figure 7:**
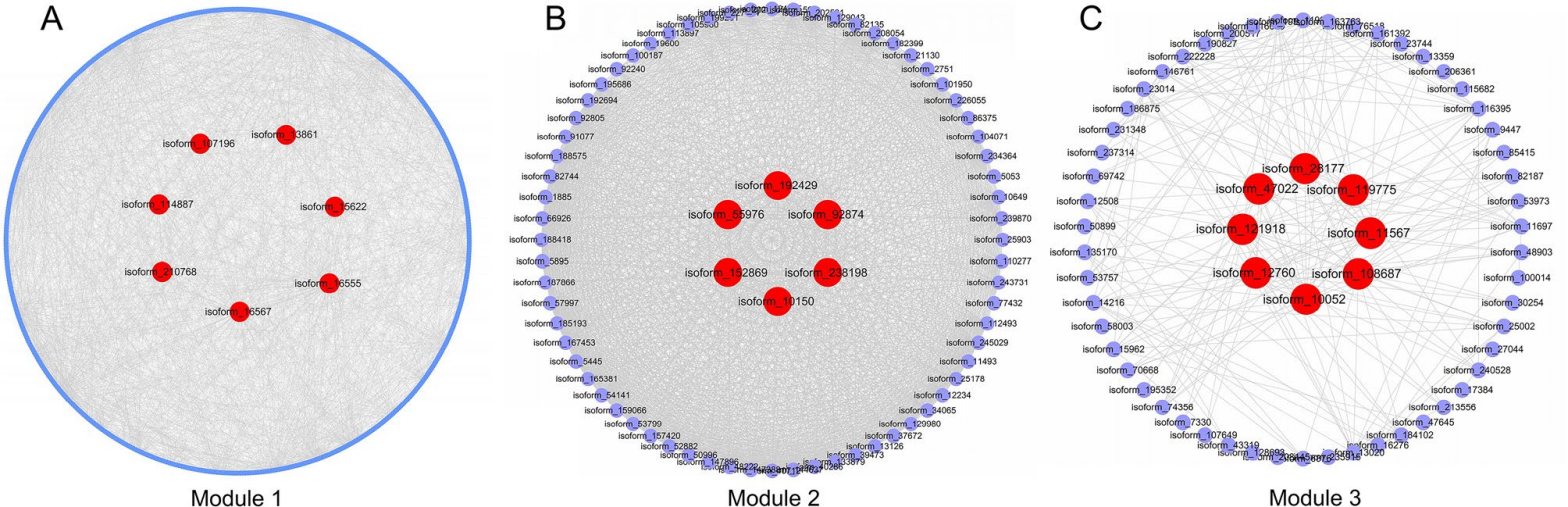
Co-expression network analysis of core light deficiency responsive modules (Cytoscape Version 3.8.2, https://cytoscape.org/). (**A**) Co-expression network of Module 1. Eight hub genes were identified in this module. (**B**) Co-expression network of Module 2. There are seven hub genes detected in this module. (**C**) Co-expression network of Module 3. Six hub genes were found in this module.

To identify hub genes in the three modules, genes with a weight parameter over 0.4 were analyzed and visualized through Cytoscape 3.8.2 to construct interaction networks (Fig. 7). We identified 6, 7 and 8 hub genes in module 1, module 2 and module 3 respectively via integrated analysis results of MCODE, cytoHubba and Centiscape2.2 in Cytoscape3.8.2 (Table 2). Isoform_16555 (Anthesis Pomoting Factor 1, *MsAPF1*), isoform_15622 (DUF1644 domain-containing protein, *MsSIZ1*), isoform_210768 (Acyl-CoA N-acyltransferase protein, *MsGNAT6*), isoform_13861 (Detoxification 21, *MsHMP21*), isoform_16567 (Mitogen-activated protein kinase 10, *MsCXIP4*), and isoform_107196 (Unknown) were identified as hub genes in module 1, indicating that these genes have significant functions in Amino acids and nucleic acids metabolism under weak light. In module 2, isoform_10150 (Beta-glucosidase 18, *MsBGL18*), isoform_92874 (Basic 7S globulin, *MsBg7S*), isoform_192429 (Cytochrome P450 710A11, *MsCYP710A11*), isoform_238198 (Transcription factor TGA2.2, *MsTGA2*), isoform_55976 and isoform_152869 (Pathogenesis-related protein P2, *MsPR4*) might play central roles in carbon fixation related pathways in response to light deficiency.

**Table 2:** The hub genes detected in three modules.

Modules	Gene ID	Gene name	Arabidopsis orthologs	Predicted functions
Module 1	Isoform_16555	*MsAPF1*	AT5G66240.1	Anthesis pomoting factor 1
	Isoform_15622	*MsSIZ1*	AT3G25910.1	DUF1644 domain-containing protein
	Isoform_210768	*MsGNAT6*	AT2G06025.5	Acyl-CoA N-acyltransferase protein
	Isoform_13861	*MsHMP21*	AT1G33110.1	Detoxifiction 21
	Isoform_114887	*MsMAPK10*	AT4G21970.1	Mitogen-activated protein kinase 10
	Isoform_16567	*MsCXIP4*	AT2G28910.2	Zinc finger protein
	Isoform_107196	*unknown*	AT1G23440.1	Unknown
Module 2	Isoform_10150	*MsBGL18*	AT1G61820.1	Beta-glucosidase 18
	Isoform_92874	*MsBg7S*	AT1G03220.1	Basic 7S globulin
	Isoform_192429	*MsCYP710A11*	AT2G34500.1	Cytochrome P450 710A11
	Isoform_238198	*MsTGA2*	AT1G68640.1	Transcription factor TGA2.2
	Isoform_55976	*unknown*	AT5G57123.1	Unknown
	Isoform_152869	*MsPR4*	AT3G04720.1	Pathogenesis-related protein P2
Module 3	Isoform_10052	*MsFLA15*	AT3G52370.1	FAS1 domain-containing protein
	Isoform_12760	*MsUGT73C7*	AT3G53160.1	UDP-rhamnose: rhamnosyltransferase 1
	Isoform_121918	*MsUGT91C1*	AT5G49690	UDP-rhamnose: rhamnosyltransferase 1
	Isoform_108687	*MsSBT3*	AT1G66220.1	Subtilisin-like protein protease SBT3.3
	Isoform_11567	*MsFLA17*	AT5G06390.1	FAS1 domain-containing protein
	Isoform_28177	*MsLECRK-V.1*	AT1G70110.1	L-type lectin-domain-containing protein
	Isoform_119775	*MsFMN*	AT4G27270.1	NADPH-dependent FMN reductase
	Isoform_47022	*MsGHL*	AT4G31500.1	Geraniol 8-hydroxylase-like protein

In module 3, isoform_10052 (FAS1 domain-containing protein, *MsFLA15*), isoform_12760 (UDP-rhamnose:rhamnosyltransferase1, *MsUGT73C7*), isoform_121918 (UDP-rhamnose:rhamnosyltransferase1, *MsUGT71C1*), isoform_108687 (Subtilisin-like protein protease SBT3.3, *MsSBT3*), isoform_11567 (FAS1 domain-containing protein, *MsFLA17*), isoform_28177 (L-type lectin-domain-containing protein, *MsLECRK-V.1*), isoform_119775 (NADPH-dependent FMN reductase, *MsFMN*) and isoform_47022 (Geraniol 8-hydroxylase-like protein, *MsGHL*) were identified as hub genes, suggesting that these genes exert vital functions in photosynthesis in *M. sinostellata* under low light conditions (Table S3).

**Figure 8:**
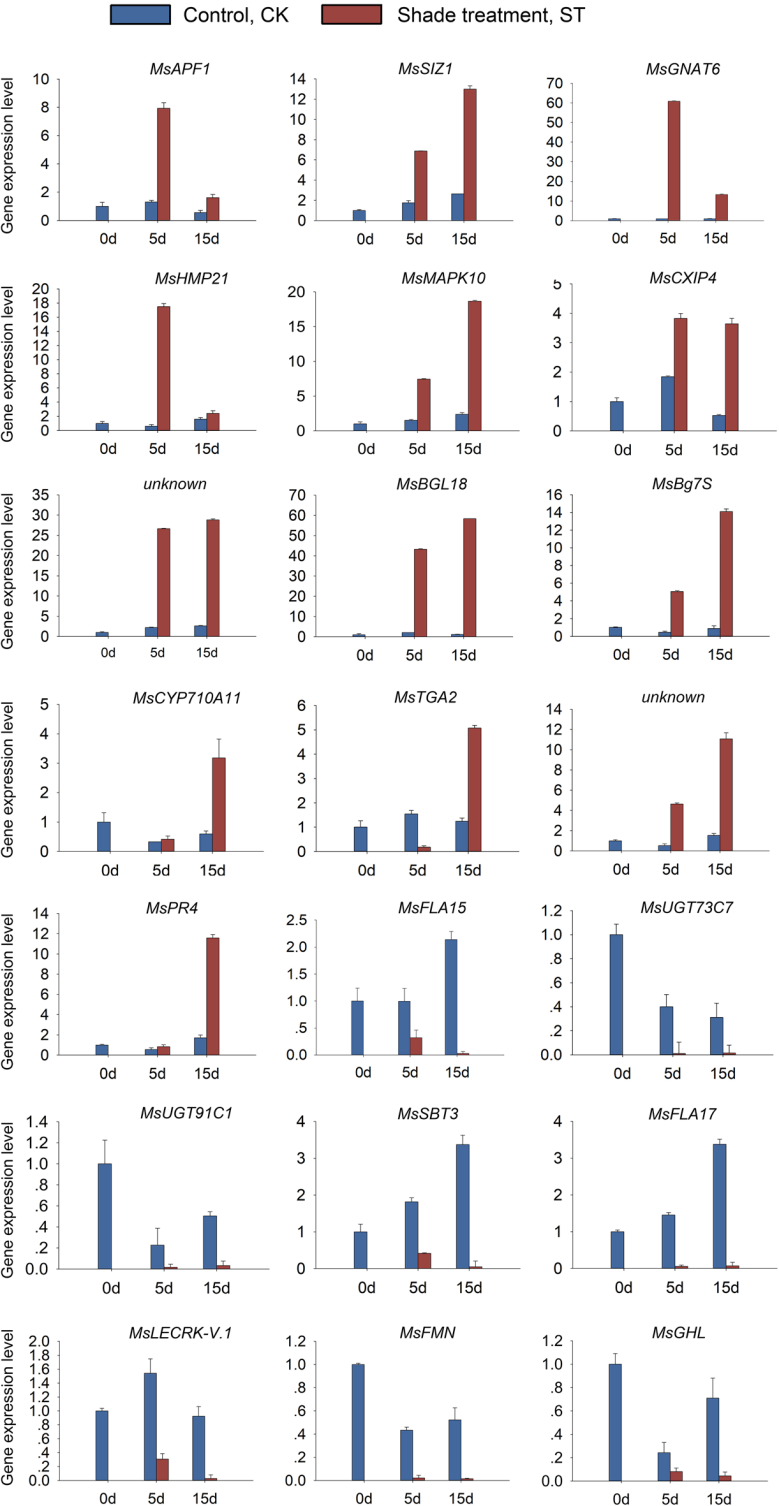
Relative expression of 21 hub genes in *M. sinostellata* under weak light and control conditions (GraphPad Prism8.0, https://www.graphpad.com). Data are the means of three biological replicates and three technical replicates. The 2^-ΔΔct^ method was employed to conduct the gene differential expression analysis.

To verify the expression of 21 hub genes under light deficiency treatment after 0d, 5d and 15d in *M. sinostellata*, quantitative reverse-transcription PCR (RT-qPCR) was performed. These results suggested that the expression level of all the hub genes were significantly altered during light deficiency treatment, which indicating that these genes were all light deficiency-responsive (Fig. 8). Interestingly, hub genes in module 1 and module 2 were all significantly up regulated during, while 8 hub genes in module 3 were all down regulated under light deficiency in *M. sinostellata*.

## Discussion

Canopy shade is a major abiotic stress that affect plants growth and reproduction in the wild. In this study, we found that shading caused light deficiency impaired photosynthesis capacity, enhanced carbon and amino acid metabolism, and flowering related pathways of *M. sinostellata* through WGCNA of transcriptome data. Light deficiency has significant impact on morphology of *M. sinostellta.* Leaves and seedlings of *M. sinostellta* gradually wilt under light deficiency treatment, and severe leaf abscission was observed, which was distinct from low light responses of module plants such as Arabidopsis^48^ and rice^46^. Photosynthesis is essential for plant growth and development, and key photosynthetic indicators *Pn* and *Fv/Fm* were both decreased significantly in *M. sinostellta*^42^.

The transcriptome analysis results confirmed these results subsequently. Genes in module 3 were all down-regulated and mostly involved in photosynthesis related pathways (Fig. 6E,F), indicating that its photosynthesis was inhibited. The suppressed photosynthesis under weak light also have been reported in various plants, including Arabidopsis^49^, cucumber^24^, maize^50^, etc. The most direct effect of impaired photosynthesis is the decreased production of glucose, followed by blocked carbon mechanism, and finally impact plant growth^51^. More importantly, glucose and its secondary metabolites sucrose and fructan are the main soluable sugar to maintain cell membrane and turgor under adverse stress^52^. Various studies have found that low light decreased content and expression of regulated genes of soluble sugars, superoxide dismutase (SOD) and peroxidase (POD) in cucumber^53^, *Olea europaea*^54^, *M. sinostellata*^7^, showing that their stress tolerance ability weakened. However, these genes mainly located in the downstream of stress tolerance molecular mechanism in *M. sinostellata*, and the hub genes in its upstream network remains obscure. In module 3, eight hub genes were identified and all regulating stress tolerance related pathways. *MsUGT73C7* (isoform_12760) and *MsUGT91C1* (isoform_121918) belongs to UDP-glucose: glycosyltransferase (UGT) family, most of which can be induced by abiotic stresses^55^. *MsUGT73C7* and *MsUGT91C1* reduced the expression of superoxide dismutase synthesis genes (isoform_15962), the function of which is to protect plants under stress by scavenging reactive oxygen and nitrogen species. Lectin-domain-containing protein and NADPH-dependent FMN reductase were reported to have essential roles in stress response^56,57^. *MsLECRK-V.1* (Lectin-domain-containing protein, isoform_28177) was predicted to down regulate the expression of *MsFMN* (NAD(P)H dehydrogenase, isoform_119775), which both able to regulate plant basal resistance^58,59^. FAS1 domain-containing proteins have important function in plant development and abiotic stress response^60^, among which *MsFLA15* (isoform_10052) and *MsFLA17* (isoform_11567) can both repress the expression of *MsFMN*. One of the vital functions of *MsFMN* is to impact utilization efficiency of photosynthetic products and plant cell wall synthesis via inhibiting the expression of cellulose synthase synthesis genes (isoform_48903), which is also an important factor to cope with various stresses^61^. Subtilisin-like protein protease and Geraniol 8-hydroxylase-like protein is associated with plant pathogen resistance and MAPK signaling pathways, respectively^62,63^. *MsSBT3* (Subtilisin-like protein protease, isoform_108687) can attenuate the expression of the *MsGHL* (Geraniol 8-hydroxylase-like protein, isoform_47022) under low light condition. These results verified that the stress tolerance ability in *M. sinostellata* under light deficiency depressed, which are essential for plant adaptation and survival.

In contrast, the expression levels of genes in module 1 and module 2 were all induced, which mainly regulating amino acid metabolism and carbon metabolism, respectively. As amino acid metabolism is strongly correlated with carbon metabolism^64^, the expression patterns of module 1 and module 2 were quite similar. Generally, the decrease in glucose production due to the inhibited photosynthesis under light deficiency will block the starch and sucrose metabolism and further carbon metabolism, such as peanut^8^ and wheat^65^. While shade tolerant plants can maintain high photosynthesis efficiency and carbon metabolism to support their life activities under light deficiency due to lengthy adaption and evolution^66^. In shade sensitive soybean, enhancing photosynthesis as well as carbon metabolism via silicon treatment can improve its growth under weak light condition^8^. Surprisingly, in *M. sinostellata*, although photosynthesis related genes inhibited under light deficiency, the genes in module 2 related to starch and sucrose metabolism and other carbon metabolism were significantly induced. The expressions of coding genes of principal enzymes in starch and sucrose metabolism including sucrose synthase and sorbitol dehydrogenase were all activated (Table S5). One theory is that low light intensity impact plant growth via a negative imbalance in carbon metabolism^67^. Owing to the enhanced starch and sucrose metabolism, the carbon imbalance state in *M. sinostellata* under weak light was much worse. The six hub genes identified in this module make this carbon metabolic molecular interacting network clearer. Basic 7S globulin is multifunctional and have essential roles in starch and sucrose pathways^68^. Beta-glucosidase 18 (*MsBGL18*) up-regulated the expression of *MsBg7S* (Basic 7S globulin, isoform_92874) and *MsCYP710A11* (Cytochrome P450 710A11, isoform_192429) in the sucrose and starch metabolism pathway in *M. sinostellata*. *MsBGL18* can also controls starch and sucrose metabolism through beta-glucosidase in *M. sinostellata*, which homology was reported involved in various pathways, including activation of chemical defense compounds, phytohormones, and metabolites^69^. Interestingly, *MsPR4* (Pathogenesis-related protein, isoform_152869) regulated key enzymes such as beta-glucosidase, beta-amylase and trehalose 6-phosphate synthase to promote the metabolism of sucrose and starch under shade. Unknown protein (isoform_55976) is unique in *M. sinostellata* was also predicted to regulate the metabolism of sucrose and starch via activating the activity of beta-glucosidase and threonine aldolase.

The environmental stimuli and alternation of metabolic states can impact plant flowering^70^. Interestingly, the effect of shading on flowering period is variant. Low R/FR ratio under vegetative shade accelerated flowering in Arabidopsis^71^ but delayed flowering in *Medicago sativa*^72^. In addition, shading caused low light intensity induced flowering related genes in peanut^8^, while postponed flowering process of *Odontonema strictum*^73^ and *Paeonia lactiflora*^11^. The expression of seven hub genes in module 1 were all increased, and mostly controlled flowering related genes, showing that flowering was promoted in *M. sinostellata* under light deficiency. DUF1644 domain-containing proteins are abiotic stress responsive in rice^74^ and Anthesis pomoting factor 1 (*AtARF1*) have essential roles in plant flowering in Arabidopsis^75^. In *M. sinostellata*, abiotic stress responsive *MsSIZ1* (DUF1644 domain-containing protein, isoform_15622)^74^ up regulated the expression of Anthesis pomoting factor *MsAPF1* (isoform_16555), which has vital function in flowering. *MsHMP21* (Detoxification 21 isoform X1, isoform_13861) enhanced the expression of important flowering-time gene Constans (isoform_25713 and isoform_21721) together with *MsAPF1*. Acyl-CoA N-acyltransferase protein was reported regulating the reproductive growth and flower bud differentiation in *Hordeum vulgare*^76^. Hub gene isoform_107196 (Unknown) was predicted to induce *MsGNAT6* (Acyl-CoA N-acyltransferase protein, isoform_210768) in *M. sinostellata*.

In general, light deficiency impaired photosynthesis, stress tolerance, and accelerated carbon metabolism and flowering in *M. sinostellata*. When the photosynthesis capacity is damaged, the enhancement in carbon metabolism exacerbated the consuming of plant carbon reservoir. Also, for perennial plants, early flowering was harmful for accumulation of reserves for resume growth in the next season^72^. Therefore, *M. sinostellata* is hyper-sensitive to light deficiency, which might be the main cause that limiting its distribution and population renewal in forests. Hub genes isoform_107196 in module 1 and isoform_55976 in module 2 were unique to *M. sinostellata*, and their functions will be investigated in our future work. This study provides new insight into light deficiency response mechanism in *M. sinostellata* and laid a firm foundation for further protection and conservation of Magnolia and other shade sensitive woody plants.

## Methods

### Plant material and experimental design

The *M. sinostellata* seedlings used in this study were collected by Yamei Shen’s research group, Zhejiang Agriculture & Forestry University, Hangzhou, Zhejiang Province, China (latitude 30°26′ N, longitude119°73′ E) and cultivated in Qingshan Lake Garden Center, Hangzhou, Zhejiang Province, China (latitude 30°25′ N, longitude119°81′ E), which both have been approved by Forestry Bureau of Zhejiang Province, P. R. China. In addition, experimental research on *M. sinostellata* was strictly comply with the IUCN Policy Statement on Research Involving Species at Risk of Extinction and the Convention on the Trade in Endangered Species of Wild Fauna and Flora. The seedlings were placed in artificial climate room (luminance 1400 ± 30 μmol m^−2^ s^ 1^, photoperiod 14 h light, temperature 25 ± 2 °C, humidity 40–60%) in Zhejiang Agriculture & Forestry University throughout the experiment. In order to simulate the closed canopy of forest, the shade experimental set-up was built with three layers of black shade net (25% full light, luminance 350 ± 30 μmol m^−2^ s^−1^) and bamboo poles. The control group seedlings were unsheltered (100% full light, luminance 1400 ± 30 μmol m^−2^ s^−1^), and the other conditions remained unchanged. There were no leaves or stems overlapping between plants, each treatment had 3 replicates. Plants were subjected to light deficiency condition for 5 (LT-D5), and 15 days (LT-D15) or kept in the control condition at indicated time points (CK-D5; CK-D15). To analysis dynamic changes of gene expression and reduce error, samples of 0 day (M-D0) used in this study were evenly collected from leave samples of control and treated groups of 0 day. These samples were frozen in liquid nitrogen and stored at − 80 °C for further experiments and RNA-seq analyses. At sampling, collections were performed from 3 plants, and each sample collection was repeated 3 times for biological replicates.

### Transcriptome sequencing and data analysis

To identify shade responsive genes, RNA sequencing was performed to analyze transcriptome gene expression profiles in 15 samples. These 15 samples were divided into five groups, including three control groups (M-D0, CK-D5 and CK-D15) and two light deficiency-treated groups (LT-D5 and LT-D15). Total RNA of 15 samples were proceed by mRNA enrichment method or rRNA removal method. The purified RNA was fragmented with the interrupted buffer and reversed with random N6 primer, and then synthesized into cDNA two-strand to form double-stranded DNA. The ends of synthetic double-stranded DNA are filled in and the 5'end is phosphorylated. The 3'end forms a sticky end with an ‘A’ protruding, and then a bubbly linker with a protruding ‘T’ on the 3'end is connected. The ligation product is amplified by PCR with specific primers. The PCR product is heat-denatured into single-stranded, and then the single-stranded DNA is circularized with a bridge primer to obtain a single-stranded circular DNA library. DNBSEQ platform was employed to sequence the libraries. The R package (edgeR v3.16) was employed to identify the differentially expressed genes (DEGs) between weak light-treated and control samples. Genes fulfilled stringent criteria were identified as DEGs (fold-change > 2 and q value < 0.05, with false discovery rate (FDR) less than 0.05). The function and involved pathways of the DEGs were classified according to the GO and KEGG^77^ annotation results and official classification. Phyper function in R software was used for enrichment analysis.

### Weighted gene co-expression network construction and hub genes detection

The weighted gene co-expression network was constructed using WGCNA package in R software to further analysis gene functions and contributing pathways in response to light deficiency in *M. sinotellata*. The 4734 core DEGs of 15 samples were used to construct this network. The similarity matrix was calculated by identifying the Pearson correlation coefficient between all gene pairs. The correlation matrix was transformed by soft-thresholding process to mimic the scale-free topology. The adjacency matrix was converted into a topological overlap matrix (TOM), and all coding sequences were hierarchically clustered by TOM similarity algorithm. The co-expression gene modules of the gene dendrogram were detected by the dynamic tree cut method, which using a height-cut less than 0.22. The module membership (MM) and the significance gene (GS) were calculated and used to confirm the distinguished modules. Module network visualization was performed by Cytoscape 3.8.2 with a cut-off of weight parameter set at 0.4. MCODE, cytoHubba and Centiscape2.2 in Cytoscape3.8.2 were used to identify hub genes.

### RT-qPCR analysis of hub genes expression

Total RNA was extracted from the leaves of *M. sinostellata* in treated and control groups of 0 d, 5 d and 15 d using ultra-clean polysaccharide and phenol plant RNA purification kit. 1 μg total RNA was converted to first-strand cDNA using the Prime Script RT master Mix. RT-qPCR were performed with Light Cycler 480 II (Roche) using BCG qPCR Master Mix. Results were analyzed with the software Light Cycler 480 SW 1.5.1. Experiments was performed under the following conditions: 95 °C for 30 s; 95 °C for 5 s and 58 °C for 30 s. A total of 40 cycles were performed. After the end of the program, melting curves were generated (65–95 °C, 0.2 °C increment). Relative gene expression was calculated using 2^−∆∆Ct^ method and *M. sinostellata EF1-α* was employed as the reference gene^5^. DNA primers used are listed in Table S4. Each sample testing was repeated at 3 times.

### Statistical analysis

Statistical analyses and graphing of venn diagrams, GO and KEGG classification^77^ and enrichment plots, diagrams of WGCNA modules, eigengene expression pattern of modules were performed using R software (version 4.0.3, www.gnu.org/software/r/). Hub genes in these three modules were visualized through Cytoscape (Version 3.8.2, https://cytoscape.org/). GraphPad Prism (GraphPad Prism8.0, https://www.graphpad.com) software were used for statistical analyses and bar charts plotting of RT-qPCR analysis of hub genes.

### Ethical approval

*Magnolia sinostellata* seedlings collected from Qingshan Lake Garden Center in Hangzhou, Zhejiang Province, China, have both been approved by Forestry Bureau of Zhejiang Province, P. R. China. In addition, experimental research on *M. sinostellata* was strictly comply with the IUCN Policy Statement on Research Involving Species at Risk of Extinction and the Convention on the Trade in Endangered Species of Wild Fauna and Flora.

## Supplementary Information

Supplementary Figure 1.

Supplementary Figure 2.

Supplementary Table 1.

Supplementary Table 2.

Supplementary Table 3.

Supplementary Table 4.

Supplementary Table 5.
