# Supplementary material for: Identification of potential light deficiency response regulators in endangered species Magnolia sinostellata
Source: Sci Rep. 2022 Dec 29;12:22536. doi: 10.1038/s41598-022-25393-x (PMC9800573; doi:10.1038/s41598-022-25393-x)
Supplement: Supplementary file 1 — Supplementary Figure 1. [file 41598_2022_25393_MOESM1_ESM.docx]

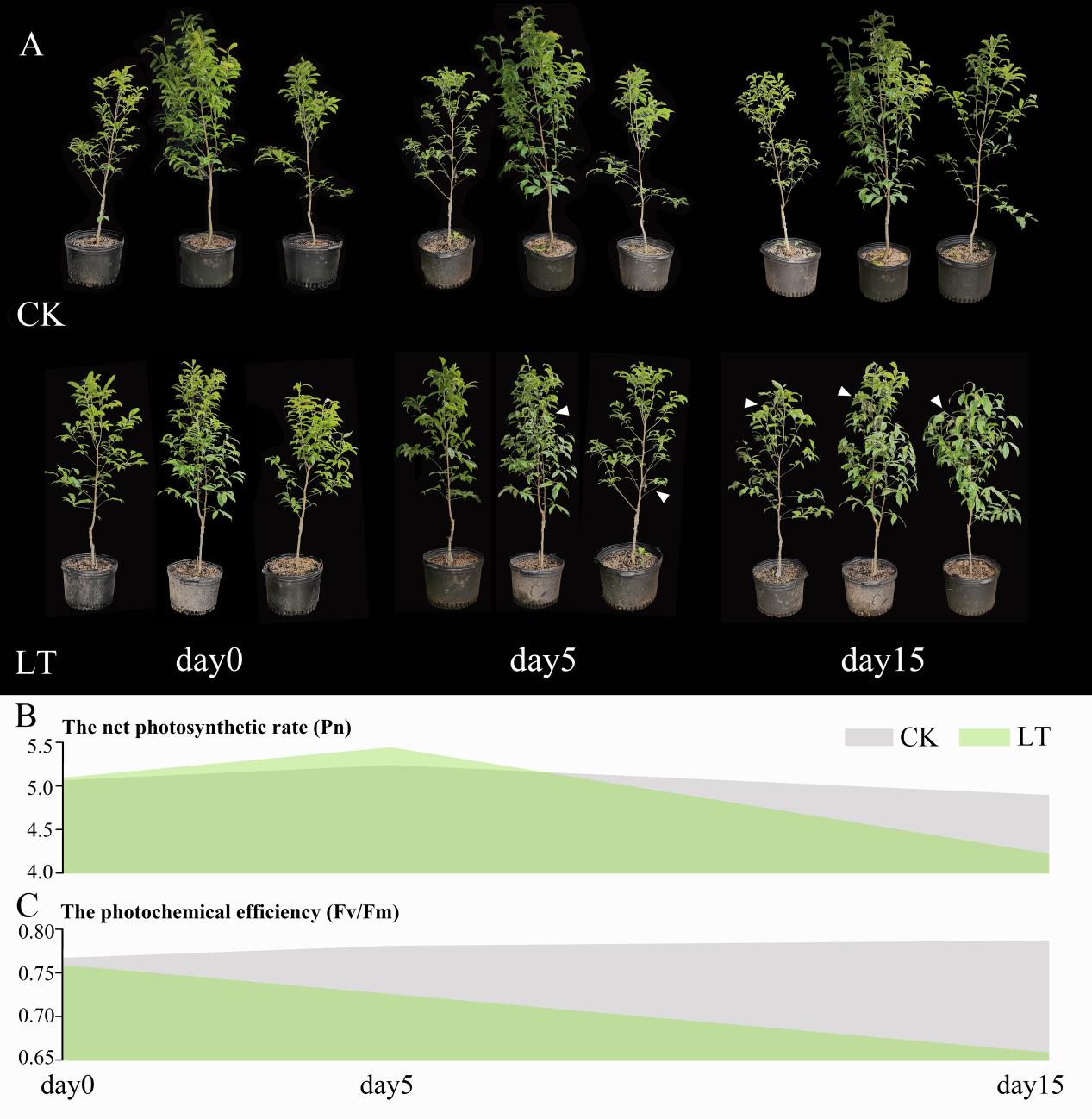


**Figure S1. Morphological and physiological changes in *M. sinostellata* under light deficiency condition**

**A.** Morphological changes of *M. sinostellata* seedlings under light deficiency condition. **B.** The net photosynthetic rate (Pn) of *M. sinostellata* during the experiment. **C.** The photochemical efficiency (Fv/Fm) of *M. sinostellata* during the experiment.
