# Supplementary figures and images for: Identification of potential light deficiency response regulators in endangered species Magnolia sinostellata
Source: Sci Rep. 2022 Dec 29;12:22536. doi: 10.1038/s41598-022-25393-x (PMC9800573; doi:10.1038/s41598-022-25393-x)

**Figure S2.** Heat map of a sample-to-sample Pearson correlation


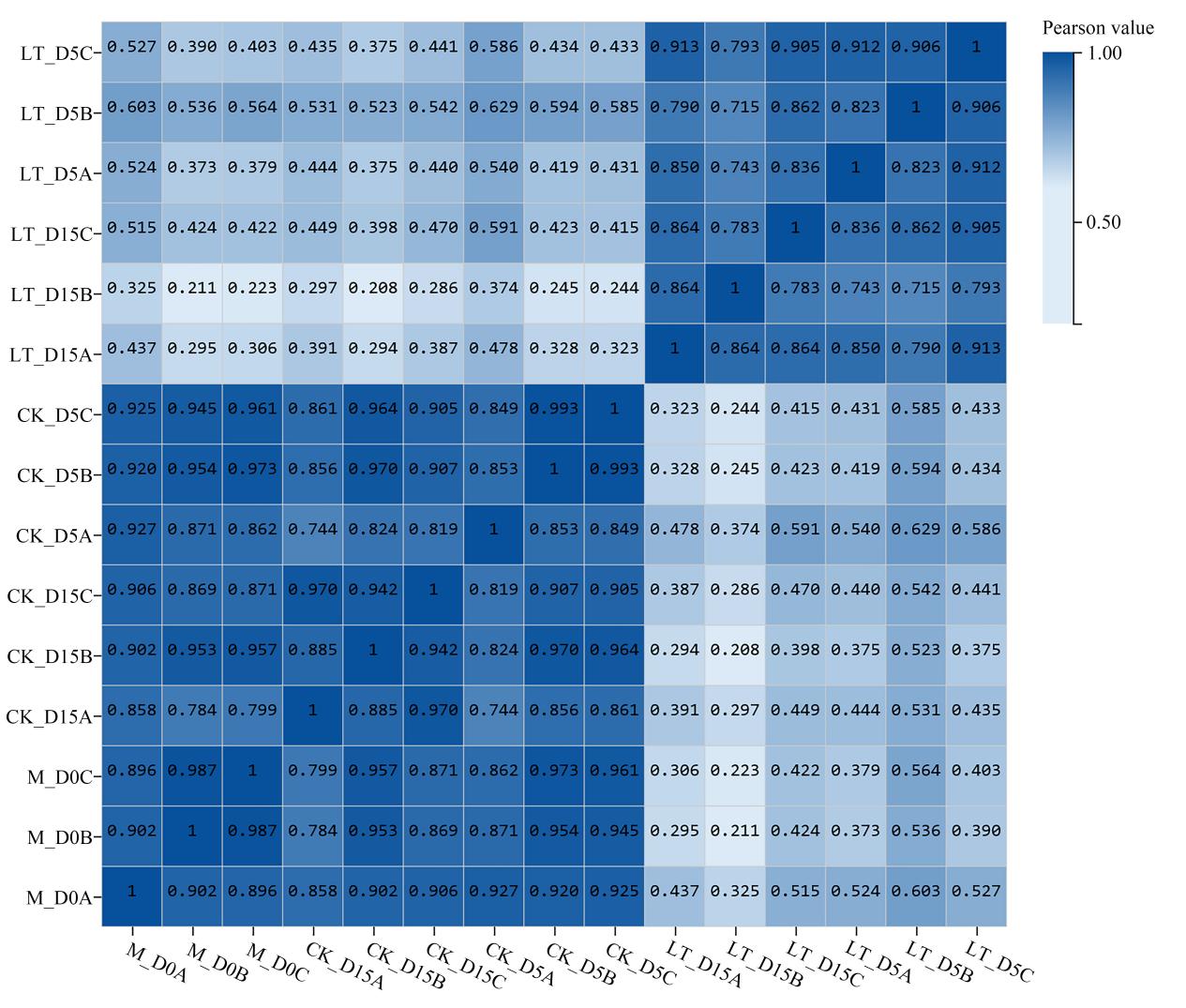

Supplement: Supplementary file 2 — Supplementary Figure 2. [file 41598_2022_25393_MOESM2_ESM.docx]
