# Supplementary material for: Identification of potential light deficiency response regulators in endangered species Magnolia sinostellata
Source: Sci Rep. 2022 Dec 29;12:22536. doi: 10.1038/s41598-022-25393-x (PMC9800573; doi:10.1038/s41598-022-25393-x)
Supplement: Supplementary file 3 — Supplementary Table 1. [file 41598_2022_25393_MOESM3_ESM.docx]

**Table S2.** Details of five datasets of *M.sinostellata*

| **Group** | **Sample name** | **Total Raw Reads (M)** | **Total Clean Reads (M)** | **Total Clean Bases(Gb)** | **Clean Reads Q20(%)** | **Clean Reads Q30(%)** | **Clean Reads Ratio(%)** |
| --- | --- | --- | --- | --- | --- | --- | --- |
| M_CK | M_D0A | 47.33 | 43.56 | 6.53 | 96.84 | 92.16 | 92.03 |
|  | M_D0B | 45.57 | 42.24 | 6.34 | 96.99 | 92.49 | 92.68 |
|  | M_D0C | 47.33 | 43.46 | 6.52 | 96.76 | 92 | 91.83 |
| D5CK | CK_D5A | 47.33 | 43.11 | 6.47 | 97.36 | 93.31 | 91.09 |
|  | CK_D5B | 47.33 | 43.3 | 6.49 | 96.84 | 92.19 | 91.48 |
|  | CK_D5C | 45.57 | 42.53 | 6.38 | 96.93 | 92.37 | 93.33 |
| D15CK | CK_D15A | 47.33 | 43.27 | 6.49 | 96.91 | 92.29 | 91.43 |
|  | CK_D15B | 45.57 | 41.95 | 6.29 | 96.62 | 91.65 | 92.04 |
|  | CK_D15C | 45.57 | 42.21 | 6.33 | 96.78 | 91.94 | 92.62 |
| D5ST | ST_D5A | 47.33 | 43.36 | 6.5 | 96.92 | 92.34 | 91.61 |
|  | ST_D5B | 47.33 | 43.53 | 6.53 | 97.22 | 92.98 | 91.98 |
|  | ST_D5C | 45.57 | 42.06 | 6.31 | 96.85 | 92.19 | 92.3 |
| D15ST | ST_D15A | 47.33 | 43.27 | 6.49 | 96.84 | 92.09 | 91.43 |
|  | ST_D15B | 45.57 | 41.99 | 6.3 | 96.87 | 92.19 | 92.13 |
|  | ST_D15C | 45.57 | 42.27 | 6.34 | 96.81 | 92.13 | 92.75 |
