# Supplementary material for: Identification of potential light deficiency response regulators in endangered species Magnolia sinostellata
Source: Sci Rep. 2022 Dec 29;12:22536. doi: 10.1038/s41598-022-25393-x (PMC9800573; doi:10.1038/s41598-022-25393-x)
Supplement: Supplementary file 6 — Supplementary Table 4. [file 41598_2022_25393_MOESM6_ESM.docx]

**Table S1.** Primers used for RT-qPCR analysis

| **Gene** | **Forward (Reverse) primer sequence** | **Annealing temperatureTm**（℃） |
| --- | --- | --- |
| isoform_10150 | F-CGGATCTATGAGGAAAGGAGC | 57.57 |
|  | R-TACCATCGGGCGGATAGTTTA | 55.61 |
| isoform_210768 | F-TCAATTTCTCACGGCAGCAC | 55.4 |
|  | R-AGCCAAAGATCCACCCAGTA | 55.4 |
| isoform_10052 | F-ATGGAGAAAGCCAGGTGAA | 53.01 |
|  | R-AACTGGTCCGTCGTGAGGC | 59.48 |
| isoform_13861 | F-GCGGAAGATATGGGTAGAGT | 55.4 |
|  | R-AGATGACGGTGGTAACAAGG | 55.4 |
| isoform_16555 | F-GCCTCTATGATGTCGCAAAT | 53.35 |
|  | R-GCAAGGAAAGAAGCCGCAGT | 57.45 |
| isoform_238198 | F-CTCCTATTCAATCGGAATGTAC | 53.95 |
|  | R-CAGGCTGCGTGACTTCGTGCT | 61.47 |
| isoform_92874 | F-TGATAACAATGGCTTTGGTGG | 53.66 |
|  | R-AGGCCCTACTCGTGTACTTCC | 59.52 |
| isoform_152869 | F-GTACGGCTGGACTGCCTTCT | 59.5 |
|  | R-AACCATTCCTATCGGTGTCG | 55.4 |
| isoform_192429 | F-ACAGATCGCATACATCCGTAA | 53.66 |
|  | R-CGAAGAGGTAGTTGGTGGAGA | 57.57 |
| isoform_10150 | F-CGGATCTATGAGGAAAGGAGC | 57.57 |
|  | R-TACCATCGGGCGGATAGTTTA | 55.61 |
| isoform_55976 | F-AGAGGGAGGAAACAAAGCGTC | 57.57 |
|  | R-CATCCAGACTGCGAGAAGAA | 55.4 |
| isoform_119775 | F-TTAATGGCGACGAAGGTTTA | 51.3 |
|  | R-CTTGGTTGGATGATTGGGAC | 55.4 |
| isoform_28177 | F-CTTGGTGCGACTTCTTGGC | 57.89 |
|  | R-TGACCACCTGCTCCCATTC | 57.89 |
| isoform_12760 | F-AGATCCAGCACCTGAAGAAG | 55.4 |
|  | R-GAAGACACTGAAGTAGGCACA | 55.61 |
| isoform_121918 | F-GATGGTGGATGTTGAGGGTG | 57.45 |
|  | R-TTGAAACCAGTGATGGGCTA | 53.35 |
| isoform_114887 | F-GCCGTCACAACCCAAGCAC | 59.48 |
|  | R-GCAGCAGGTTCACCTCCAT | 57.32 |
| isoform_47022 | F-GTCCCGCTCCTTATCCCACG | 61.55 |
|  | R-CTCCCTCCCGACGAACCTCT | 61.55 |
| isoform_107196-r-1 | F-TTGAGAAAGCAGGGCTATGA | 53.35 |
|  | R-CAAGAGTGACGCCACGAATT | 55.4 |
| isoform_11567 | F-ATGGAGAAAGCCAGGTGAA | 53.01 |
|  | R-CGATAACTGGTCCGTCGTG | 57.32 |
| isoform_108687 | F-TGGCTATGCTTTCCTTTCGG | 55.4 |
|  | R-CCCACCAGACTCATCGCTTG | 59.5 |
| isoform_16567 | F-GTCAAGTGCGACGACAAGG | 57.32 |
|  | R-AGCAATAATCCGCTCAATC | 50.85 |
| isoform_15622 | F-GGAGTCTGCCCGCCAGTTCA | 61.55 |
|  | R-TCCAGTCCCGCTGCCGTTCT | 61.55 |
